# Patterns of sexual dimorphism in Mexican alligator lizards, *Barisia imbricata*

**DOI:** 10.1002/ece3.455

**Published:** 2012-12-26

**Authors:** Daniel Dashevsky, Jesse M Meik, Estrella Mociño-Deloya, Kirk Setser, Sarah Schaack

**Affiliations:** 1 Department of Biology, Reed CollegePortland, Oregon; 2 Department of Biology, University of TexasArlington, Texas; 3 Departmento de Biologia Animal, Universidad de GranadaGranada, España

**Keywords:** Body size, Mexican alligator lizards, natural selection, sexual dimorphism, sexual selection

## Abstract

We compare morphological characteristics of male and female *Barisia imbricata*, Mexican alligator lizards, and find that mass, head length, coloration, incidence of scars from conspecifics, tail loss, and frequency of bearing the color/pattern of the opposite sex are all sexually dimorphic traits. Overall size (measured as snout–vent length), on the other hand, is not different between the two sexes. We use data on bite scar frequency and fecundity to evaluate competing hypotheses regarding the selective forces driving these patterns. We contend that sexual selection, acting through male-male competition, may favor larger mass and head size in males, whereas large females are likely favored by natural selection for greater fecundity. In addition, the frequency of opposite-sex patterning in males versus females may indicate that the costs of agonistic interactions among males are severe enough to allow for an alternative mating strategy. Finally, we discuss how sexual and natural selective forces may interact to drive or mask the evolution of sexually dimorphic traits.

## Introduction

Although sexual dimorphism (SD) has been observed in many organisms, quantifying trait values and considering the hypotheses that can potentially explain such differences is a major step toward identifying the causes underlying differences in morphology and behavior within and between sexes. Understanding SD is complicated because differences between male and female phenotypes can result from natural selection, sexual selection, or both (Fleming and Gross 1994; Vanhooydonck et al. 2010; Kaliontzopoulou et al. 2012). Similarly, distinct sexual or natural selective pressures acting in parallel or opposition can result in no net morphological difference between sexes. If sexual monomorphism is the result, the influence of strong selective regimes may not be apparent. In this study, we describe patterns of sexual dimorphism in the Mexican alligator lizard (*Barisia imbricata*) and consider the impact of both natural and sexual selection pressures.

Sexual size dimorphism (SSD) is particularly well-studied and has been observed in a wide variety of organisms (e.g., Ralls 1977; Carothers 1984; Fleming and Gross 1994). SSD may result from natural selection, for example for greater fecundity (leading to larger body size in females; Pincheira-Donoso and Tregenza 2011) or favoring earlier age at maturity (leading to reduced body size in either sex; Denoël et al. 2009). It is also possible that natural selection may cause SSD due to niche divergence between sexes (Shine 1989; Bolnick and Doebeli 2003). In addition, sexual selection can favor body size differences (e.g. for territory defense, combat, or mate choice; Darwin 1871; Anderson and Vitt 1990; Tokarz 1995; Pianka and Vitt 2003). It is important to note, however, that natural and sexual selection pressures are not mutually exclusive and may act in concert to influence size and other sexually dimorphic traits in lizards and other organisms (Ji et al. 2006; Vincent and Herrel 2007).

**Figure 1:**
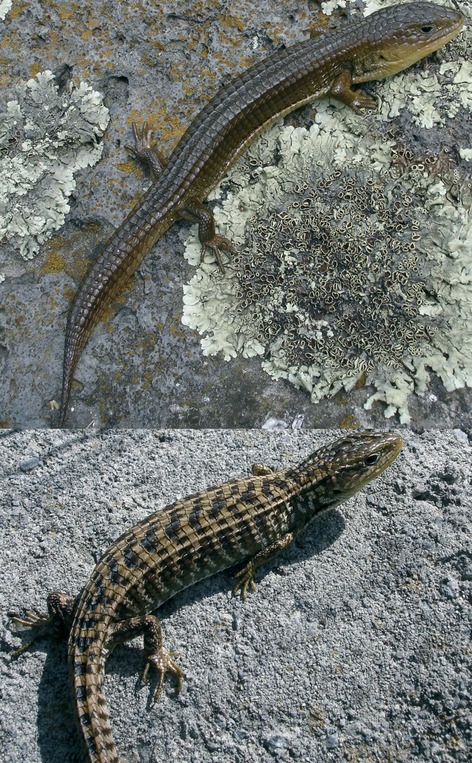
Typical color patterning for male (top) and female (bottom) *B. imbricata* (note: the male has a partially regenerated tail; photo by J. Meik).

Here, we investigate sexual dimorphism and evaluate potentially interacting natural and sexual selective pressures in a population of *Barisia imbricata*, Mexican alligator lizards. The *Barisia imbricata* (Anguidae) complex includes four species of viviparous anguid lizards, of which *B. imbricata* is the most widespread. These lizards are endemic to temperate and subtropical regions of Mexico at mid to high elevations (Tihen 1949; Guillette and Smith 1982; Good 1988; Zaldivar-Riverón et al. 2005). In most populations from the Transvolcanic axis of central Mexico, males are uniform brown or green, whereas females are brown with pale transverse bands (Guillette and Smith 1982; Zaldivar-Riverón et al. 2005; Fig. 1). In a previous study of the reproductive biology of *B. imbricata*, Guillette and Casas-Andreu (1987) found no SSD. Natural selection for increased fecundity in females, however, operating simultaneously with intrasexual selection for large males, can result in no net SSD (Cooper and Vitt 1989; Vial and Stewart 1989). Although *B. imbricata* has not previously been reported to engage in male-male combat, closely related gerrhonotine lizards do (Vial and Stewart 1989; Wicknick 1990). In this study, we measure a variety of traits in males and females, including size characters, color patterns, frequency of bite scars and caudal autonomy, and an important fitness trait, fecundity.

## Materials and Methods

The study population of *B. imbricata* was collected from an agricultural area in the valley of the Rio Lerma, near Atlacomulco, Estado de México, Mexico (elevation approx. 2500 m). Lizards were collected primarily from pastures, along fencerows, and from canal embankments. Lizards were collected during June and July 2006 and temporarily maintained in individual plastic tubs at a processing facility before being returned to the site of capture. Most individuals were released within 24 h; however, we retained gravid females until parturition (Supplemental Figure S2). For each individual, we recorded the following data: sex, snout–vent length (SVL), mass, head length, head depth, head width, snout length, injuries (any scars or bite scars that appear to be from conspecifics [Supplemental Figure S1]), color pattern (uniform brown/olive or banded, as well as whether individuals displayed the color pattern typical of the opposite sex [OSP]), whether the individual had undergone caudal autotomy (note, it was not possible to determine if an individual had shed its tail multiple times), and whether a female was gravid. Additionally, litter size was recorded after females gave birth.

We measured mass (g; to the nearest 0.1 g) with an electronic balance and took linear measurements with digital calipers (mm; to the nearest 0.1 mm). Both head width and head depth were taken at the anterior edge of the auricular openings; head length was measured from the tip of the snout to the posterior edge of the auricular opening. Snout length was measured as the distance between the anterior margin of the eye and the external nares. We determined sex by everting hemipenes and by examining the base of the tail for hemipenal bulges. Sex designation for juveniles was not always certain (i.e., hemipenes might be present but undetected). Thus, juveniles were excluded from analyses.

We calculated body length by subtracting head length from SVL. We then used body length, rather than SVL, for analyses of head size because of the direct contribution of head length to SVL. We used *t*-tests to compare means of SVL, head length, head width, head depth, snout length, and mass between males and females (gravid females were excluded from analysis of mass, see Table 1). We used chi-squared tests to determine if sex had a significant effect on the incidence of scarring, bite scarring, caudal autotomy, and OSP (see Table 1). We used linear models with a dummy variable for sex and interaction effects to test whether sex influenced relationships among continuous variables. Statistical analyses were performed using R version 2.14.1.

**Table 1:** Characters measured in *Barisia imbricata* (*n* = 29 males and 49 females) and results of statistical analyses of differences between sexes for each character using *t*-tests or chi-squared (*χ*^2^)

	Mean (±SE)					
					
Character	Male	Female	*t*	*χ*^*2*^	df	P
Snout-Vent Length (mm)	110 ± 2	108 ± 2	–0.42	***–***	76	0.7
Head Length (mm)	24.4 ± 0.6	21.3 ± 0.4	4.26	***–***	76	<0.0001
Head Width (mm)	18.1 ± 0.5	15.3 ± 0.2	5.66	***–***	76	<0.0001
Head Depth (mm)	13.8 ± 0.5	11.9 ± 0.2	4.14	***–***	76	<0.0001
Snout Length (mm)	9.4 ± 0.2	8.5 ± 0.1	3.88	***–***	76	0.0002
Mass (g)	27 ± 2	18 ± 2	3.09	***–***	49	0.003
Any Scars	17%	10%	**–**	0.81	1	0.4
Bite Scars	10%	0%	**–**	5.94	1	0.01
Caudal Autotomy	93%	71%	**–**	5.25	1	0.02
Opposite Sex Patterning	24%	6%	**–**	5.29	1	0.02

## Results

**Figure 2:**
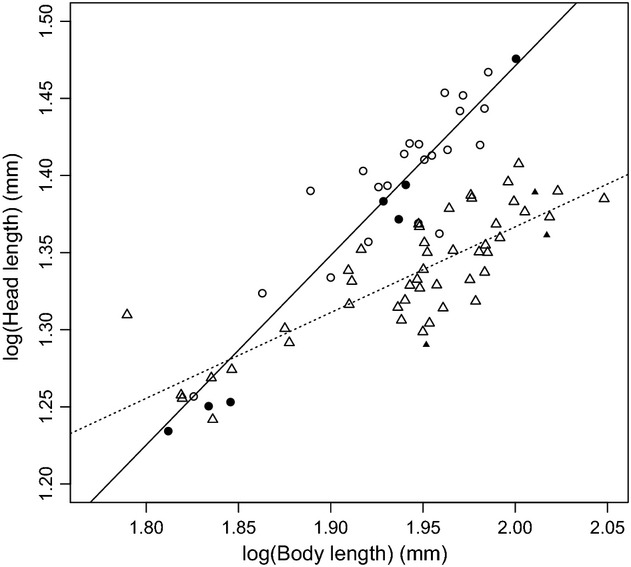
Head length as a function of body length for males (◯, solid line) and females (△, small dashes). Filled symbols represent individuals who exhibited OSP.

**Figure 3:**
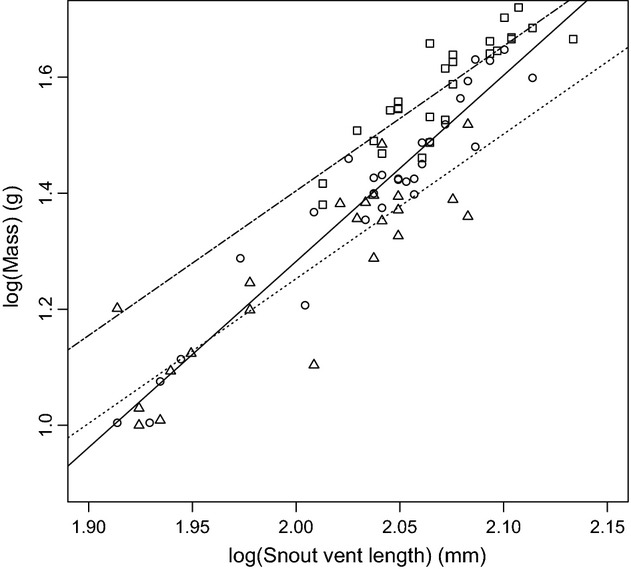
Mass as a function of SVL for males (◯, solid line), gravid females (□, alternating dashes), and non-gravid females (△, small dashes).

We collected 85 individuals: 29 males, 49 females, and 7 juveniles (three of which were too young to determine their sex). The largest male was 130 mm SVL, the largest female was 136 mm SVL, and the smallest gravid female was 103 mm SVL. There was no difference in SVL between the sexes, but head length, head depth, head width, snout length, and mass were larger in males than females (see Table 1; gravid females were removed from analyses of mass). Males have proportionally longer heads than females (see Fig. 2); the best model for predicting head length includes both body length and mass (*R*^2^ = 0.8866; *F* = 142.7, df = 4 and 73). As with head length, the mass of males increases more than that of females for a given increase in SVL (Fig. 3; *R*^2^ = 0.9147, *F* = 154.4, df = 5 and 72). Males also exhibit larger head width, head depth, and snout length with regard to body length (Supplemental Figure S3).

**Figure 4:**
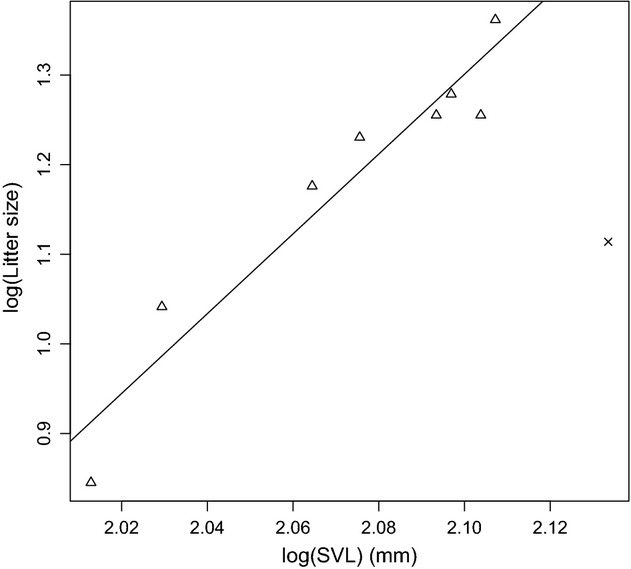
The relationship between litter size and SVL for captured female *B. imbricata* (*n* = 8; one outlier [x] was excluded from the regression because this mother had begun giving birth prior to capture).

The frequency of bite scarring was higher in males (3 of 29) than in females (0 of 49), however, scarring of all types was not significantly different (5 of 29 and 5 of 49 for males and females, respectively; Table 1). Caudal autotomy was more frequent among males (27 of 29) than females (35 of 49). The frequency of opposite sex patterning (OSP) was higher for males (7 of 29) than for females (3 of 49). There was also a trend for OSP individuals to have shorter heads (*n* = 10, *t* = −1.916, *P* = 0.059). Scarring and tail loss were not significantly correlated (*χ*^2^=2.96, df = 1, *P* = 0.08). Linear models showed that there was no effect of SVL, which can be used as a proxy for age, on the likelihood of any scarring, bite scarring, caudal autotomy, or OSP. Female SVL and litter size was strongly positively correlated (Fig. 4; *R*^2^ = 0.9188, *F* = 67.88, df = 1 and 6).

## Discussion

Male and female *B. imbricata* are sexually dimorphic for a number of traits. Morphologically, males have larger heads and greater masses than females, but overall length (SVL) does not differ between the sexes. In addition, males exhibit a higher incidence of bite scars and caudal autotomy (tail loss) as well as a higher frequency of opposite sex patterning. The differences between sexes in these traits suggest that a number of selective forces may be acting. Such forces may be interacting with each other, as well as with unrelated genetic and developmental constraints (Fairbairn 2007; Mank et al. 2010). Understanding how these factors determine size, shape, and coloration in this system may provide insight into how sexual dimorphism arises in other organisms (Badyaev 2002).

### Male-male combat and sexual dimorphism

The increased incidence of bite scars among males is likely due to intrasexual agonistic encounters. This is supported by the observation that (1) only males showed evidence of bite scars (Supplemental Figure S1), in contrast with scarring in general which was not different between the sexes and (2) the higher incidence of tail loss in males. Vitt et al. (1974) found evidence for tail loss due to male-male encounters in *Sceloporus magister*. Attacking rivals' tails may be a good strategy during intrasexual competition if tail loss decreases the likelihood of mating, as found by Martín and Salvador (1993). An alternative explanation is intersexual differences in predation pressure; however, the frequency of CA has not been correlated with predation pressure in several species of lizards (Jaksic and Greene 1984; Bateman and Fleming 2009).

Differences in bite scar frequency may be related to the sexual dimorphism observed in head length. Several studies have found a correlation between male-male combat and sexually dimorphic head size in lizards, potentially due to the advantage greater bite force confers during competition (e.g., Carothers 1984; Herrel et al. 2010; Vanhooydonck et al. 2010). However, large head length in males could also be explained by intraspecific niche partitioning (e.g., males and females may consume different prey thereby reducing competition for resources). Although previous studies have not supported a role for niche partitioning in sexual dimorphism in lizards (Perry 1996; Herrel et al. 1999; Butler et al. 2000), evaluating this hypothesis would require data on foraging behavior and/or diet for this species.

We observed a significant difference in mass between the sexes, despite finding no such difference in SVL. Although this could be due to a simple life-history trade-off (e.g., females divert resources from growth to reproduction), it could also reflect the larger mass of male heads. Lizard skulls contain many bony elements and males can have hypertrophied jaw musculature and increased ossification (Anderson and Vitt 1990; Pianka and Vitt 2003), which are traits that may be selected for if agonistic interactions among males include biting (Husak et al. 2009). Taken together, the patterns we observed in bite scars, caudal autotomy, head length, and mass suggest that male *B. imbricata* experience positive selective pressure on body size due to male-male combat.

### Mimicry as an alternative mating strategy

The higher frequency of OSP in males (more males resemble females than vice versa) could be explained by several hypotheses: (1) it is easier for a male to adopt or exhibit a female pattern during development than the opposite, (2) there is stronger negative selection on OSP in females than males or (3) due to their resemblance to females, OSP males avoid costly intrasexual competition. We do not have data to address hypothesis 1, but it is relevant to note that, in the sampled population, neonate patterns are more similar to adult males than adult females, suggesting that female patterning requires additional factors to develop (Supplemental Figure S2). Intriguingly, in other populations of the *B. imbricata* complex, and for gerrhonotine lizards in general, neonates have been reported to exhibit female patterning (Zaldivar-Riverón et al. 2005). One of the only mechanisms to explain hypothesis 2 would be if female color pattern in *B. imbricata* is under sexual selection, but the same trait in males is not subject to selection. Although male mate choice is not common in lizards, it has been observed in *Sceloporous virgatus* (Weiss et al. 2009). However, unlike *S. virgatus*, female *B. imbricata* do not have bright coloration or patterns that fluctuate based on their reproductive cycle, so it is unlikely that male mate choice is a driving factor in female patterning.

Evidence for hypothesis 3 (female mimicry as an alternative mating strategy) has been observed in other lizards (Sinervo and Lively 1996; Whiting et al. 2009), but has not been previously seen in *B. imbricata*. Evidence from other populations of *B. imbricata*, and from other species in the complex, suggests that sexual dichromatism may be developmentally and evolutionarily labile (Zaldivar-Riverón et al. 2005). Although further study would be necessary to evaluate this hypothesis, the correlation between OSP and smaller head length suggests that males that resemble females do so for multiple traits (Fig. 2). This may not, however, be due to selection favoring an alternative mating strategy because genes controlling color patterning and head size could be linked or co-regulated in this species. Behavioral data and/or morphological data from additional populations could help distinguish among these competing hypotheses to explain why opposite sex patterning is more common in males.

### Parallel natural and sexual selection

Although males seem to face sexual selection for large head size, *B. imbricata* do not display sexual dimorphism in overall body size (SVL), a pattern observed in other species (e.g., Dubey et al. 2011). One explanation for this is the strong positive relationship between SVL and litter size observed in females (Fig. 4). This relationship indicates that large body size may be selected for in female *B. imbricata* due to increased fecundity, a pattern observed in many species (e.g., Shine 1989; Honěk 1993; Corl et al. 2010). Our results contrast with those obtained from the only study of female reproduction in *B. imbricata* (Guillette and Casas-Andreu 1987). Their study, conducted using individuals from a higher elevation population (3000–3400 m vs. 2500 m), found no correlation between SVL and litter size in females, but also reported smaller average litter sizes (7 vs. 16 in this study) and a lower range of SVL (78–125 mm vs. 103–136 mm in this study). The differences between the two studies suggest geographic variation in female reproductive traits.

In conclusion, natural selection for increased body size in females (due to increased fecundity of large females) may be paralleled by sexual selection in males for larger body size (due to the advantage gained during intraspecific agonistic behavior). The occurrence of such parallel or interacting selective forces may obscure sexual dimorphism in some traits. This, and the evidence that intraspecific and interspecific interactions may exert different levels of selection pressure (Calsbeek and Cox 2010), therefore requires more detailed studies to ascertain the various selective forces operating on male and female morphology and development within and between species (Cox et al. 2003).

## Supporting Information

**Figure S1.** A male *Barisia imbricata* with a scar from being bitten by a conspecific (photo by J. Meik).

**Figure S2.** A mother *Barisia imbricata* along with a brood of neonates delivered in captivity. Note the solid pattern, typical of males, is exhibited by all neonates regardless of sex in contrast with the color pattern typical of females (mother) which emerges later in development (photo by J. Meik).

**Figure S3.** A) Head length (*R*^2^ = 0.8866; *F* = 142.7, df = 4 and 73), B) head width (*R*^2^ = 0.8676; *F* = 119.6, df = 4 and 73), C) head depth (*R*^2^ = 0.8465; *F* = 100.6, df = 4 and 73), and D) snout length (*R*^2^ = 0.7591; *F* = 57.52, df = 4 and 73) as functions of BL for males (^, solid line) and females (Δ, small dashes). Filled symbols represent individuals who exhibited OSP.
