# Supplementary material for: Patterns of sexual dimorphism in Mexican alligator lizards, Barisia imbricata
Source: Ecol Evol. 2012 Dec 26;3(2):255–61. doi: 10.1002/ece3.455 (PMC3586635; doi:10.1002/ece3.455)
Supplement: Supplementary file 1 [file ece30003-0255-SD1.docx]

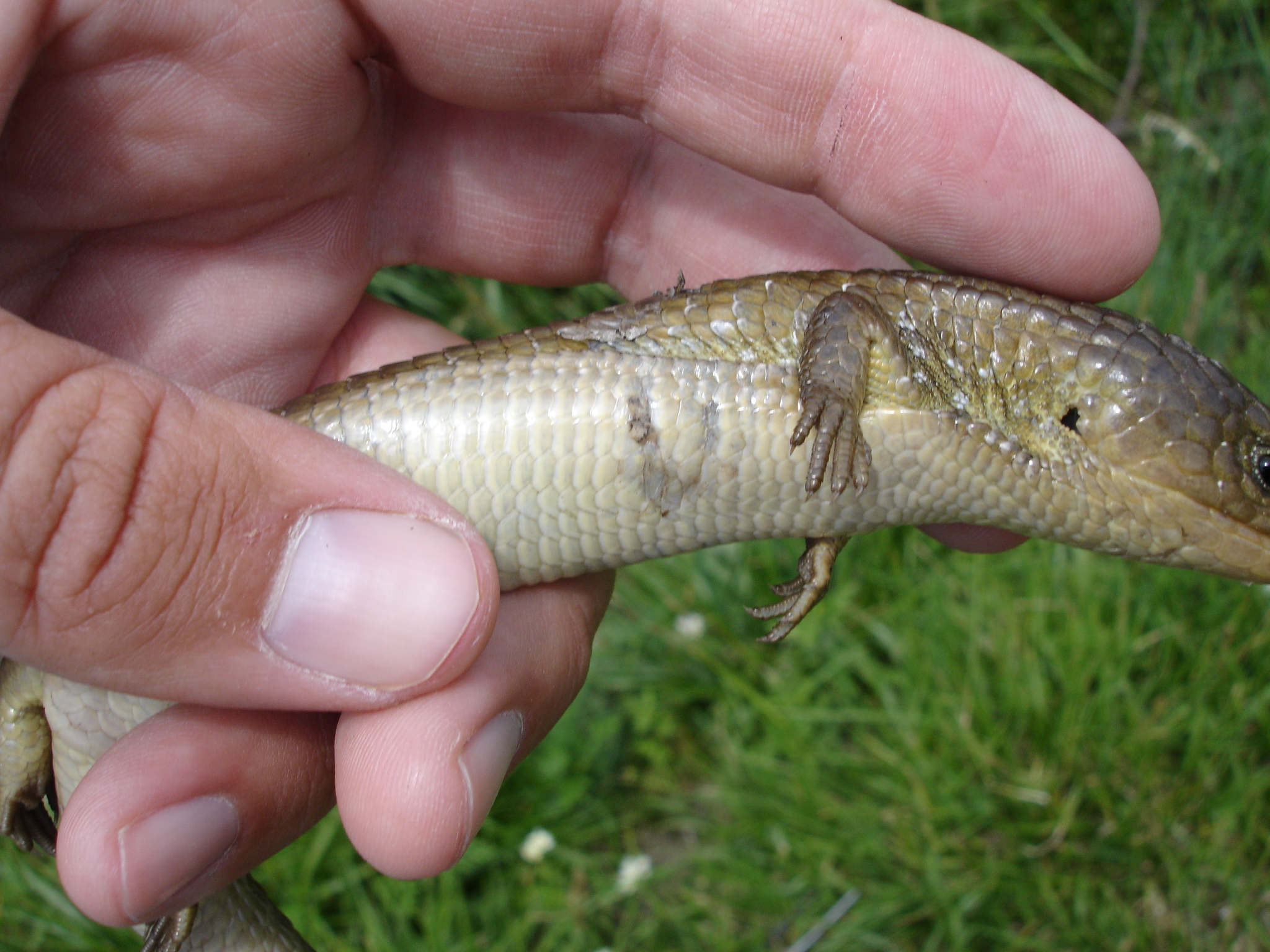
**Supplemental Figure S1.** A male *B. imbricata* with a scar from being bitten by a conspecific (photo by J. Meik).
