# Supplementary material for: Patterns of sexual dimorphism in Mexican alligator lizards, Barisia imbricata
Source: Ecol Evol. 2012 Dec 26;3(2):255–61. doi: 10.1002/ece3.455 (PMC3586635; doi:10.1002/ece3.455)
Supplement: Supplementary file 2 [file ece30003-0255-SD2.docx]

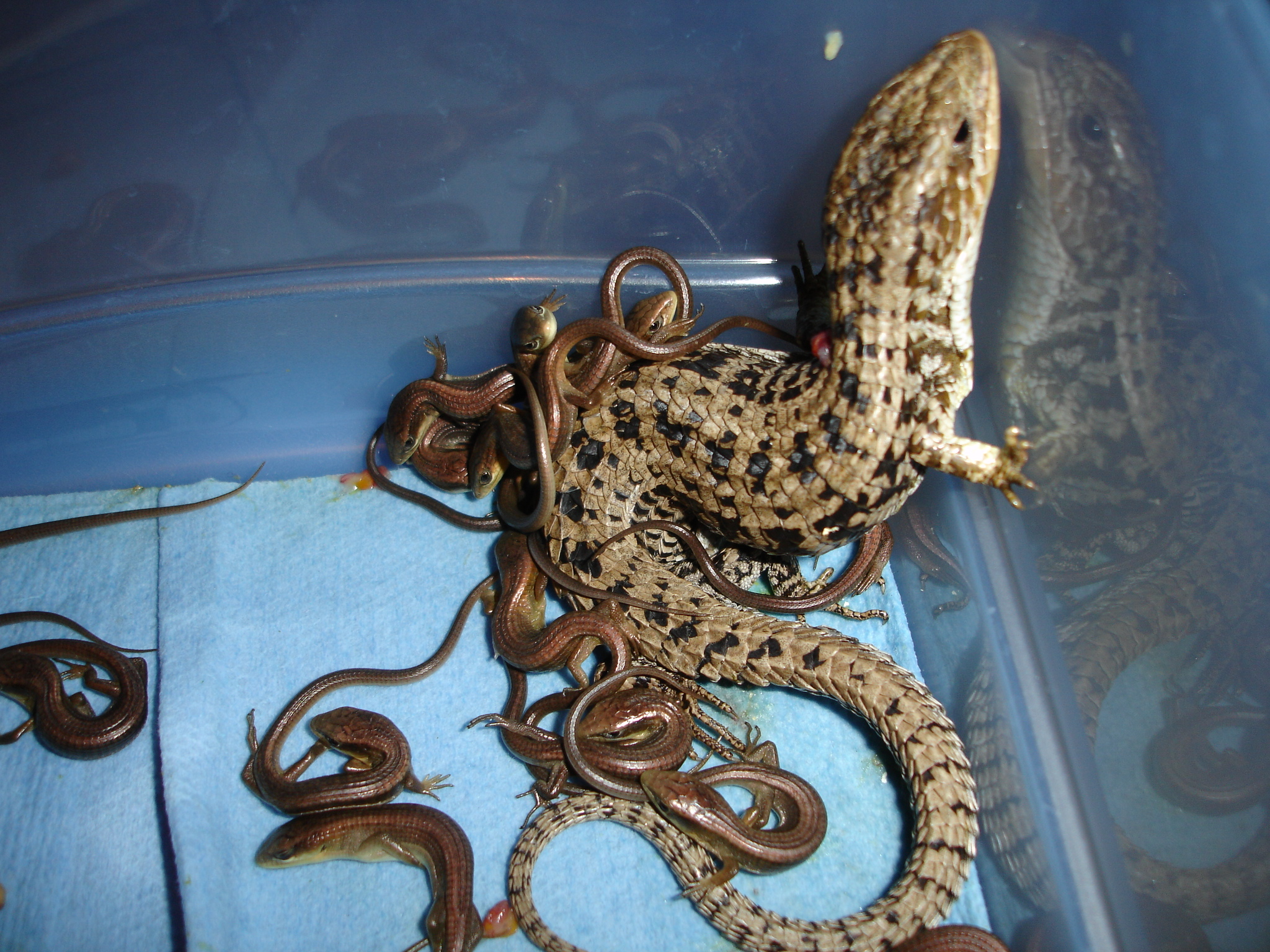
**Supplemental Figure S2.** A mother *B. imbricata* along with a brood of neonates delivered in captivity. Note the solid pattern, typical of males, is exhibited by all neonates regardless of sex in contrast with the color pattern typical of females (mother) which emerges later in development (photo by J. Meik).
