# Supplementary material for: Patterns of sexual dimorphism in Mexican alligator lizards, Barisia imbricata
Source: Ecol Evol. 2012 Dec 26;3(2):255–61. doi: 10.1002/ece3.455 (PMC3586635; doi:10.1002/ece3.455)
Supplement: Supplementary file 3 [file ece30003-0255-SD3.docx]

**Supplemental Figure S3.** A) Head length (*R*^2^ = 0.8866; *F* = 142.7, df = 4 and 73), B) head width (*R*^2^ = 0.8676; *F* = 119.6, df = 4 and 73), C) head depth (*R*^2^ = 0.8465; *F* = 100.6, df = 4 and 73), and D) snout length (*R*^2^ = 0.7591; *F* = 57.52, df = 4 and 73) as functions of BL for males (◯, solid line) and females (△, small dashes). Filled symbols represent individuals who exhibited OSP.
